# Fear of Recurrence of Atrial Fibrillation: Translating a Cancer Fear Model to the Atrial Fibrillation Patient Experience

**DOI:** 10.3389/fpsyt.2022.915327

**Published:** 2022-07-04

**Authors:** Scarlett Anthony, Rebecca Harrell, Caroline Martin, Taylor Hawkins, Saleen Khan, Aditi Naniwadekar, Samuel F. Sears

**Affiliations:** ^1^Department of Psychology, East Carolina University, Greenville, NC, United States; ^2^Department of Cardiovascular Sciences, East Carolina University, Greenville, NC, United States

**Keywords:** atrial fibrillation, cardiac electrophysiology, cardiac psychology, fear of recurrence, health anxiety

## Abstract

**Background:**

Atrial fibrillation occurs when rapid and disorganized electrical signals cause the atria in the heart to beat irregularly and is associated with an increased risk for stroke. Despite medical advancements, first and second line atrial fibrillation treatments exhibit significant recurrence rates. Because of this, atrial fibrillation patients often experience disease-specific fears that are not routinely assessed and targeted in clinical management. Fear of recurrence models in cancer research and other cardiac-specific fears have paved the way for a patient-centric approach to disease intervention.

**Purpose:**

Clinical assessment focused solely on the taxonomy of anxiety disorders may miss key components unique to the atrial fibrillation patient experience. An anxiety disorder diagnosis in the presence of an arrhythmia could be misleading and ultimately fail to address patient needs. Modeled from the cancer literature, providers may benefit from a broader disease specific conceptualization for AF patients that differs from a general DSM-5 diagnosis.

**Aims:**

The objectives of this paper are: (1) to review the medical aspects of atrial fibrillation, (2) to examine the comparability of fear of recurrence concept from cancer literature to the atrial fibrillation patient, and (3) to suggest considerations of these novel concepts in patient care.

**Future Directions:**

Increased understanding of fear of recurrence among atrial fibrillation patients aims to define and assess fear of recurrence components, determine treatment targets, and ultimately improve patient outcomes.

## Introduction

Consultation liaison psychiatrists and psychologists are often called to the bedside or clinic room of a medical patient coping with multiple health challenges. The use of the Diagnostic and Statistical Manual of Mental Disorders, Fifth Edition [DSM-5, (1)] provides the blueprint for examining psychological disorders and making diagnoses. As the evidence base grows for the psychological aspects of chronic diseases, mental health professionals are faced with evaluating patients with significant anxieties that are either consistent with, or part of, the disease process. This scenario presents a significant challenge for providers as the manifestations of disease-specific fears may be difficult to diagnose and, therefore, unresponsive to traditional treatment recommendations for DSM-5-defined anxiety disorders. Even so, illness-related anxieties surrounding fears of symptoms or conditions worsening are common for individuals with chronic conditions (2). Patients presenting with cardiac arrhythmias may experience spontaneous palpitations, shortness of breath, exercise intolerance, and other discomforts that mimic anxiety disorders. Nonetheless, the diagnosis of an anxiety disorder in the presence of an arrhythmia with similar symptoms could be misleading.

Psycho-oncology literature has rapidly deployed cancer-specific constructs in an effort to understand the patient experience of the unique aspects of living with cancer. The juxtaposition of cancer and cardiac disease constructs to describe the disease experience may provide complementary insights, as both conditions have significant impacts on perceived health and longevity, disease burden, life interruption, multiple treatment modalities, and recurrent follow-up care. Both disease states have disease-specific fears that involve patients navigating the health care system, while feeling poorly, and managing future threats. As a result, perceptions and expectations of the future become an important aspect for both disease states–specifically around the phenomenon of fear of recurrence (FoR).

Consulting psychiatrists and psychologists may increasingly be referred patients with AF. The purposes of this paper are: 1) to review the medical aspects of atrial fibrillation, 2) to examine the comparability of FoR concept from cancer literature to the AF patient, and 3) to apply these novel concepts in patient care.

## Medical Aspects of AF

Atrial fibrillation (AF) occurs when rapid and disorganized electrical signals cause the atria in the heart to irregularly beat, restricting blood flow from the atria to the ventricles (3). Over time, AF can decrease the heart's pumping ability which can lead to heart failure and is also associated with an increased risk of stroke or systemic embolism (4). In the United States alone, roughly 3 to 6 million people are impacted by AF with domestic and global prevalence expected to increase to 12.1 million by 2030 and up to 17.9 million by 2050 in Europe (5–7). Individuals aged 65 years and older are most likely to be diagnosed with AF, however, it can occur among younger patients especially if precipitated by other cardiac risk factors (e.g., hypertension, premorbid cardiovascular conditions, lifestyle and behavioral risk factors) or familial predisposition (8). Before the age of 75, men are more often diagnosed with AF compared to women (9), although women exhibit elevated risk of adverse events and AF incidence and burden (10–12). Similar patterns are demonstrated among Black and Brown patient populations with lower overall AF prevalence, but greater risk for heightened symptom burden and adverse events (13).

Presentation of AF can also vary significantly among individuals, and the presentations are categorized as paroxysmal AF (e.g., AF episodes occur spontaneously for less than a week and are often responsive to first- or second-line treatments), persistent AF (e.g., episodes occur for longer than a week and require additional intervention), and permanent AF (e.g., chronic irregular heart rhythm often unresponsive to repeated interventions). Typical symptoms reported by AF patients across all presentations include heart palpitations, lightheadedness/dizziness, tiredness/fatigue, shortness of breath, and chest pain (14). Interestingly, as much as 39% of AF patients report never having experienced any AF symptoms (15). Irrespective of symptom reporting, worsened disease progression (e.g., moving from paroxysmal to persistent to permanent presentations) can occur among AF patients with poorly managed co-morbid conditions, significant psychological distress, and behavioral risk factors among the most vulnerable (10).

The comprehensive treatment of atrial fibrillation requires multidisciplinary collaboration and includes primordial anti-arrhythmic and rate control agents, catheter ablation, and lifestyle management (16). The first-line treatment option for patients requires consideration of risk factors, duration of atrial fibrillation, left atrial fibrosis, and other individual factors (17, 18). Further, the introduction of direct oral anticoagulants and the addition of left atrial appendage occlusion devices have increased the effectiveness and safety profile of stroke prophylaxis in AF. Recurrence of atrial fibrillation while taking antiarrhythmic agents is close to 40–70%, compared to 20–50% with ablation (18, 19). Initial antiarrhythmic drug therapy can reduce recurrent AF episodes for some patients; however, catheter ablation interventions have demonstrated effectiveness at reducing recurrence risk (19, 20). Even so, ablative interventions do not significantly reduce risk of adverse events when compared to pharmacological treatments (20), and in 1 year following ablation and cardioversion, up to 40% of people can still have recurrence [despite anti-arrhythmic medications (21)].

Despite all of the advancements in the treatment paradigm of atrial fibrillation, some patients can remain focused on AF recurrence with each of these approaches (22, 23). While some patients may never experience recurrence of an AF episode after the initial treatment, variables such as obesity, thyroid abnormalities, alcohol consumption and obstructive sleep apnea have all been closely linked to increased probability of recurrence of AF (24). Given the notable probability for recurrent AF episodes following first and second-line treatments and the behavioral link, identifying the role of recurrence possibility for patients is critical. Thus, contemporary management of atrial fibrillation is multi-pronged: reduction of stroke risk, prevention of AF incidents with medical, surgical, or electrical means, and reduction of recurrence to promote comprehensive symptom relief with aggressive risk factor modifications (16, 19, 22).

## Fear of Recurrence: Psychological and Behavioral Aspects

FoR refers to specific anxiety among medical patients with episodic disease bouts of varying intensity and causality that are resistant to forecast. In the US, cancer accounted for nearly 10 million deaths in 2020 (25) compared to approximately 26,000 caused by AF (26). Despite this difference in mortality, the possibility for adverse events (e.g., stroke and premature death) is also prominent in AF patient populations (5), and, as such, it appears that the emotional dimensions and behavioral implications of these conditions share some similarities. Similar to cancer patients in remission, AF patients may exhibit significant fears related to the possibility of recurrence. Specifically, AF symptoms and recurring episodes due to high rates of AF recurrence are anticipated by patients (27).

While this concept has yet to be translated to understanding the AF patient experience, both FoR models in cancer research and prior research regarding other well-defined cardiac-specific fears (e.g., ICD shock anxiety) have taken a two-factor approach to understanding patient fears–examining both *fear of antecedents and consequences* (28, 29). Similarly, (30) have recently utilized a cancer model as a framework for understanding recurrence fears among patients following acute coronary syndromes (ACS). Birk and colleagues are currently examining its impact on medication adherence and physical activity engagement in a randomized trial targeting patients fears vs. usual care. While this work will help better inform the feasibility of transferring cancer-informed ideas about FoR to cardiac populations and how to treat it, conceptualization of AF patient fears maybe more specific and centered around the AF patient experience. Clinical care of FoR among cancer patients spans reliable assessment, psychoeducation, signs and symptoms, and cognitive behavioral therapy (CBT) strategies for risk reduction and follow-up (31). Similar action-oriented interventions can be integrated into AF patient care to better target specific fears and produce considerable patient benefit.

### Fear of Recurrence Among Cancer Patients

Within populations of cancer survivors, it is estimated that roughly 30–70% of patients endorse having moderate to severe levels of recurrence fears that can persist for years following diagnosis (32). Models of FoR generally involve antecedent and consequence-focused specific fears of cancer relapse. These models have proposed that patient perception of physical sensations and identification of symptoms often serve as a trigger for elevated fears that can result in increased symptom reporting, significant psychological distress, feelings of hopelessness, and diminished quality of life (32, 33). In addition, FoR among cancer survivors has been linked to increased depressive symptoms, frequent rumination, and impairment in functioning (34). Fear of recurrence has also been associated with heightened death anxiety and intrusive and threatening thoughts about death and dying (35, 36). Patients who have experienced disease recurrence exhibit heightened fear of disease progression and fear of loss of autonomy and related emotional distress (37).

Behavioral impacts of FoR generally occur on a spectrum–with some patients becoming hypervigilant about their health (i.e., over-utilization of health care, constantly monitoring symptoms and displaying control-seeking behaviors), while others become avoidant [i.e., missing routine appointments, avoiding necessary testing, non-adherence to medications and treatment regimens; (32, 34, 38)]. Differences in FoR in cancer survivors have also emerged in regard to gender with women experiencing FoR at higher rates than men (39). In addition, patients with lower education, decreased income, and younger age are also more likely to experience FoR following cancer remission. Even so, a pattern exists between high FoR and diminished engagement in health behaviors, such as physical activities like walking or running (40). The body of literature for cancer patients is substantial, and many of these specific fears (and manifestations) are mirrored in the AF patient experience.

### Fear of Recurrence in Atrial Fibrillation Patients

FoR in AF populations can also be understood as a two-factor concept-composed of both patient fears around antecedents (e.g., fear of activities that could trigger an AF episode) and consequences (e.g., fear of what may happen if an AF episode occurs). In other words, specific to this population, FoR can be conceptualized as cognitions and behaviors associated with either fear of triggering an AF episode or fear of the potential consequences of AF recurrence. Interestingly, recent research has demonstrated that only alcohol use was a reliable trigger in a study utilizing patient selected triggers for AF (41), suggesting that commonly held ideas about the triggers of caffeine, reduced sleep, exercise, lying on the left side, dehydration, large meals, and other customized triggers were not associated with the occurrence of AF.

While the concept of FoR can be theorized into two distinct components, it is important to recognize that FoR is multidimensional and encompasses patient-perceived possible triggers, emotions, thoughts, behavioral reactions, and coping strategies (see Table 1). Additional value or utility of FoR includes that cardiac providers would likely be comfortable reviewing the chances and the impact of AF recurrence. Identifying patients with high FoR for AF would allow for more targeted patient education, whereas addressing more general anxiety would likely not be as responsive to patient education. Moreover, recent constructs, such as confidence in living with AF (42), provide cardiac clinics with other patient centered metrics to evaluate positive or desirable aspects of the patient experience as well. Taken together, increased theory and metrics of the AF patient experience can address both fears and the initiation of empowerment.

**Table 1:** Signs, symptoms, and steps for identifying and targeting AF patient fears.

**Components of fear of recurrence**	**Manifestations (avoidance, catastrophizing, hypervigilance)**	**Assessment/Recommendations**
Fear of physical activity (exercise, sexual activity, emotional reactivity)	♢ Avoidance of normal activities ♢ Body listening ♢ Decreased emotions ♢ Relationship difficulties ♢ Worsened medical severity	♢ Cardiac rehab is a safe and manageable option for AF patients. It may increase comfort and has been proven to decrease arrhythmic burden for some AF patients (43). ♢ Psychoeducation on the importance of physical activity may be necessary. ♢ AF patients often have unique fears regarding physical activity. Stress management techniques may be beneficial to reduce anxiety and gain confidence. ♢ Some AF patients become deconditioned as they resign from typical activities. It may be helpful to schedule pleasant activities to boost mood and aid in re-engagement.
Fear of death	♢ Increased healthcare utilization ♢ Catastrophizing ♢ Withdrawal/avoidance (e.g., missing routine appointments, non-adherence) ♢ Decreased QOL	♢ Psychoeducation on one's prognosis may alleviate some distorted beliefs and provide reassurance. ♢ Reducing catastrophizing thinking through CBT techniques can decrease distress and improve QOL (44).
Fear of stroke (loss of independence, burden, subsequent death)	♢ Increased health-specific anxiety ♢ Caregiver concerns	♢ Education around the importance of anticoagulant therapy could better inform patients about risk of stroke (e.g., significantly lowered if patients are adherent). ♢ Taking a shared-decision making approach to discussing anticoagulant therapy (and AF-specific treatment plans) could help the patient feel as if they are in the driver's seat of their health decisions (and possibly re-establish patient confidence and reduce worry around loss of independence).
Fear of medical consequences (finances, procedures, medical severity)	♢ Financial Stress ♢ Apprehension ♢ Avoidance of seeking healthcare to save money ♢ Non-compliance	♢ Reviewing social determinants of health and financial support to ensure equitable healthcare delivery. ♢ Utilization of payment assistance programs to offset costs of copayments and/or prescription medications (45).

### Commonalities of Patient Psychological Responses in AF to Cancer

The patient experience of AF symptoms has long included a curious component with a significant portion of patients (approximately 39%) reporting that they were asymptomatic (15). Objective metrics of the presence of AF allow for examination of both the objective and subjective components of AF symptoms. This research suggested that negative emotions were more strongly associated with AF symptom score than the presence of device-detected AF (46). More recently, observational studies using serial assessment of AF patients in a specialty clinic demonstrated that depression and anxiety were the best predictors of AF symptom severity and that symptom severity was associated with increased costs and healthcare utilization (47). These data further necessitate understanding the psychological aspects of AF symptom reporting and the AF patient experience.

Approximately 28–38% of AF patients experience significant depressive or anxious symptoms (11, 48), and the interrelationships between these symptoms and AF are multidimensional and complex (49). Those patients taking the anti-coagulant, warfarin, as a method for stroke prevention are also at an increased risk of depressive symptoms and dementia (50). These high rates of emotional reactivity may be attributed to AF specific fears, consequences, and demanded psychological adjustment (51). The combination of uncertainties regarding the cause, symptom presentation, treatment options, and outcomes of AF can be overwhelming for patients (52). AF on its own is not typically life threatening, however, AF increases the risk of stroke by nearly five times and is the most commonly reported fear by AF patients, and for good reason (53, 54). Those patients with both AF and history of stroke experience increased rates of medical and neurological complications and are more likely to face consequences such as disability and even death when compared to stroke patients without AF (55–57). The current assessment of fear of stroke revolves largely around physical functioning (e.g., mobility, memory and thinking, communication, etc.) and emotional impacts following the event rather than future, stroke-related fears (58). In addition to fear of stroke, death anxiety is a salient fear in those with chronic conditions such as AF (59, 60) and refers to feelings of discomfort, worry, or fear that may follow becoming aware of one's own mortality (61). Death anxiety in patients has been shown to be negatively related to quality of life; that is, those with higher levels of death anxiety often experience poorer quality of life (62). Collectively, psychological well-being can strongly impact AF symptom severity and patient healthcare utilization (63).

Similar patterns in individual and group differences identified in research regarding FoR in cancer populations may also be reflected in AF patient populations. For instance, women living with AF consistently exhibit higher rates of depression and anxiety, increased incidence of physical AF symptoms and symptom burden, and decreased quality of life when compared to their male counterparts (11, 44, 64–66). In addition, younger AF patients (i.e., <60 years old) are seemingly more impacted by health-related stress and experience depression and anxiety at higher rates than older patients (65). Patients with lower SES and diminished access to resources are at increased risk for elevated AF symptom burden and adverse events [e.g., premature death, heart failure, MI, and stroke; (67)]. Interestingly, although female patients have demonstrated diminished quality of life and worse symptoms, they are less likely to seek secondary AF treatment such as catheter ablations (68), and as such, may be particularly vulnerable to continued psychological concerns. Moreover, it is likely that women, patients of lower SES (and lower access to resources and health literacy), and younger age are also at increased risk for developing AF-specific fears contributing to worse outcomes–a notion echoed in cancer FoR literature.

### Behavioral Changes and Response in AF Patients

The importance of patient understanding and adherence to the medical regimen is common across chronic diseases and can prompt a set of changes spanning behavioral, emotional, and medical challenges (69).

For the AF patient, ongoing clinical care includes the management of the risk factors of AF, such as adherence to medical management (e.g., rate control, rhythm control, blood pressure control, and stroke prophylaxis) and engagement in risk reduction behaviors (e.g., weight management, abstinence from smoking, avoiding or minimizing alcohol consumption, and participation in regular physical activity) (70).

Even a quick review of these risk behaviors would indicate that many of these changes are indicated as part of a healthy lifestyle for all patients. The progression of AF can be associated with the presence and combination of behavioral risk factors, and young-onset individuals may even have risk factors that are maintaining their condition (71). However, the challenges for AF patients are multiplicative because existing research in both China and in Europe suggest that as few as 4% of AF patients are engaged in a fully “healthy lifestyle” (72). Some modifiable risk factors have been shown to have distinct associations with atrial fibrillation (e.g., alcohol consumption, being overweight/obese, smoking) and the cumulative impact poses significant risk for these patients [Di (73)]. Successes and failures at making these lifestyle changes often chip away at patients' perceptions of control, daily planning to engage in valued activities, and life itself (74). Many patients fear that physical activity may trigger an AF episode which initiates a behavioral avoidance pattern to exercise/exertion (75). Finding meaning in AF symptoms, trying to “stay clear” of AF episodes, and managing unpredictable and functional limits are also common behavioral responses that can translate into increased distress and worsened quality of life (76).

## Diagnostic Considerations

A common goal of AF patient care among psychiatrists and psychologists typically involves determining if an anxiety disorder diagnosis is warranted and then providing typical recommendations or a referral for the treatment of an anxiety disorder. Considering the specificity of AF patient experiences, it may not be ideal to pursue general anxiety disorders for these patients. Although some symptoms may overlap, patient concerns are often specifically related to AF symptoms and risk factors. Moreover, a simple phobia diagnosis also does not fit the phenomena well. Many AF patients' “approach” the fear of irregular heartbeat constantly by seeking information and diagnostics in an attempt to detect AF. Similar to routine care among cancer patients, psychiatrists and psychologists can assess for FoR, provide adequate medical information, discuss signs and symptoms of recurrence, normalize patient fears, implement strategies, and provide a plan for follow-up (31). AF patients could likely benefit from a similar action-oriented plan encompassing their individual fears and providing specific treatment plans as needed for the future. The activation of therapeutic interventions, such as exercise programs, alcohol reduction, and OSA management, would be expected to be exceedingly more beneficial than a diagnosis alone.

Each AF patient likely has a unique combination of the established components of FoR in AF. It is common for AF patients to experience a spectrum of disease-specific fears such as fear of exercise, fear of death, fear of stroke, and fear of other medical consequences. Table 1 consists of these components of fear of recurrence, manifestations of those fears, and recommendations for each.

Specific action plans that can be efficiently established during regularly scheduled appointments can improve patient outcomes, improve rapport, and decrease healthcare utilization for AF patients.

## Conclusion and Future Directions

The FoR model in AF is proposed to give consultation liaison psychiatrists and psychologists a broader disease-specific conceptualization related to the AF patient experience, beyond traditional anxiety disorders in AF patients in hopes to improve patient outcomes. The FoR concept in cancer has proven useful for targeting and reducing disease-specific fears among cancer patients. A similar model for AF patients may also provide utility for consultation liaison psychiatrists and psychologists to recognize and address similar fears in the AF patient. In turn, the aim is to help advance provider assessment of FoR components, possible presentations and manifestations, and specific fears that can be targeted in AF management plans. The importance of this work is highlighted in that many patients may feel misunderstood or disregarded when their fears are not recognized by providers and instead receive anxiety disorder diagnoses that are not representative of their experience (77). Overall, FoR assessment can help normalize the patient experience and provide education around rationality of fears. In addition, it can help patients balance the risk and benefits of engaging in previously feared behaviors necessary for proper AF management (e.g., physical activity). It is our belief that AF patient care involves a commitment to understanding unique patient fears outside of what can be defined by the DSM-5. We can borrow from cancer FoR assessment strategies to inform AF care by addressing disease-specific fears.

Tailored treatment therapies from FoR in cancer should also be further evaluated for applicability to help target AF patients' shared experiences. A growing number of therapeutic interventions have demonstrated effectiveness in managing FoR among cancer patients (31). Psychological interventions (e.g., CBT interventions) have been identified as efficacious treatments for alleviating FoR among cancer survivors with effects immediately following intervention and at follow-up periods. Of note, contemporary CBT interventions have demonstrated greater efficacy in treating FoR than traditional CBT interventions, indicating that treatments aimed at altering how individuals relate to inner experiences (e.g., worry, rumination, etc.) are especially advantageous (34). Similar strategies spanning behavioral, psychological, and cognitive concerns can be translated into AF-specific clinical care to improve patient outcomes. As a first step to targeted target, we are currently developing and testing a measure assessing AF FoR informed by the model presented in the current paper. This model intends to provide a blueprint for further assessment and future treatments in hopes to improve the AF experience.

## Author Contributions

SA contributed to the conceptualization with support from SS, RH, CM, SK, TH, and AN. SS, SA, and RH contributed to visualization. SS, SA, RH, CM, TH, and SK contributed to the writing—original draft. SA, SS, RH, TH, SK, and AN contributed to writing—review and editing. SS supervised the writing of this article. All authors contributed to the article and approved the submitted version.

## Conflict of Interest

SS serves as a consultant to Medtronic, Abbott, Milestone Pharmaceutical, and Zoll Medical. He has received honorarium from Medtronic, Biotronik, and Zoll Medical in the past 12 months. The remaining authors declare that the research was conducted in the absence of any commercial or financial relationships that could be construed as a potential conflict of interest.

## Publisher's Note

All claims expressed in this article are solely those of the authors and do not necessarily represent those of their affiliated organizations, or those of the publisher, the editors and the reviewers. Any product that may be evaluated in this article, or claim that may be made by its manufacturer, is not guaranteed or endorsed by the publisher.
